# Sequence-based drug-target affinity prediction using weighted graph neural networks

**DOI:** 10.1186/s12864-022-08648-9

**Published:** 2022-06-17

**Authors:** Mingjian Jiang, Shuang Wang, Shugang Zhang, Wei Zhou, Yuanyuan Zhang, Zhen Li

**Affiliations:** 1 School of Information and Control Engineering, Qingdao University of Technology, Qingdao, 266525 China; 2 College of Computer Science and Technology, China University of Petroleum, Qingdao, 266580 China; 3 College of Computer Science and Technology, Ocean University of China, Qingdao, 266100 China; 4 College of Computer Science and Technology, Qingdao University, Qingdao, 266071 China

**Keywords:** Sequence representation, Drug-protein affinity prediction, Graph neural network

## Abstract

**Background:**

Affinity prediction between molecule and protein is an important step of virtual screening, which is usually called drug-target affinity (DTA) prediction. Its accuracy directly influences the progress of drug development. Sequence-based drug-target affinity prediction can predict the affinity according to protein sequence, which is fast and can be applied to large datasets. However, due to the lack of protein structure information, the accuracy needs to be improved.

**Results:**

The proposed model which is called WGNN-DTA can be competent in drug-target affinity (DTA) and compound-protein interaction (CPI) prediction tasks. Various experiments are designed to verify the performance of the proposed method in different scenarios, which proves that WGNN-DTA has the advantages of simplicity and high accuracy. Moreover, because it does not need complex steps such as multiple sequence alignment (MSA), it has fast execution speed, and can be suitable for the screening of large databases.

**Conclusion:**

We construct protein and molecular graphs through sequence and SMILES that can effectively reflect their structures. To utilize the detail contact information of protein, graph neural network is used to extract features and predict the binding affinity based on the graphs, which is called weighted graph neural networks drug-target affinity predictor (WGNN-DTA). The proposed method has the advantages of simplicity and high accuracy.

## Background

The drug-target affinity prediction is a key task in virtual screening, which has been studied for decades. The prediction can be used to determine whether the small molecule can bind to a target, which could be further applied to screen lead compounds to speed up drug research and development. Sequence-based method and structure-based method are two commonly used methods in drug-target affinity prediction. The difference is that whether the protein structure is provided or not. For a target with known structure, we can use molecular docking and molecular dynamics simulation to predict the binding conformation and binding strength between molecule and protein, which can obtain a relatively accurate result. There are several programs that can be implemented for molecular docking, such as DOCK [1] and AutoDock [2]. However, structure-based method has many limitations. On the one hand, molecular docking requires conformational searching, which is time-consuming, and it is expensive to screen a large database containing hundreds of millions of small molecules. On the other hand, with the development of proteomics, protein sequencing is very fast, but its structure is still difficult to obtain, which means that there are still many targets without structural information. Sequence is the only information for these targets that can be used to predict binding affinities with small molecules, which is called as sequence-based affinity prediction.

Sequence-based method has always been a research hotspot in computational biology. Various neural networks have been tried to extract the feature of protein sequence and molecule SMILES (simplified molecular input line entry specification) [3]. Sequence-based method can be further divided into two types according to different demands, including compound-protein interaction (CPI) prediction and drug-target affinity (DTA) prediction. CPI prediction is a simplified DTA prediction, which is a binary classification task and could predict whether the drug can bind to the target. For example, TransformerCPI [4] uses a transformer neural network [5] to mine sequence information for the CPI prediction. Liu et al. [6] builds up highly credible negative samples and combines various sources such as chemical expression profiles, sequences information and protein functional annotations into a systematic screening framework to get the CPI prediction. However, it is need to generate many profiles for the pre-processing, which is time consuming. DeepScreen [7] is an individual predictor for a specific target using a deep convolutional neural network. NeoDTI [8] integrates various sources from heterogeneous network data and uses topology-preserving representations of drugs and targets to implement interaction prediction. Hu et al. [9] proposes a CNN-based method for drug-target interaction prediction, which takes 1D, 2D structural descriptors of drug and sequence of protein as the network inputs. To achieve a more accurate prediction, decision tree and kernel ridge regression [10] are used for feature dimensionality reduction and ensemble learning.

Different from CPI prediction, DTA prediction can predict the detailed binding affinity between drug and target, it is usually a regression task and has aroused great interest in recent years. For instance, DeepDTA [11] is made up of two convolutional neural networks (CNN), which are used to learn the latent vectors of protein sequences and drug SMILES respectively to predict their affinity. WideDTA [12] improves DeepDTA by adding two extra CNNs to represent the additional protein domains and motifs (PDM) and ligand maximum common substructures (LMCS). DeepPurpose [13] utilizes two encoders to represents the SMILES and sequence, which are composed of CNN, recurrent neural network (RNN) and Transformer. GANsDTA [14] uses a semi-supervised generative adversarial networks (GAN) for feature extraction to predict binding affinity. Shim et al. [15] proposes a method based on CNN, which involves the outer products between column vectors of two similarity matrices for the drugs and targets to predict the affinity. For molecule representation, molecular fingerprinting has always been an effective way, which includes extended connectivity fingerprints (ECFPs) [16], atom-environment fingerprints (MOLPRINT2D) [17] and molecular access system keys (MACCS) [18]. Because the obtained fingerprinting is a vector composed of 0 and 1, it can be easily learned by neural networks. Moreover, MSTG [19] utilizes a substructure tree to describe molecule, which is used for generating molecule in drug design and achieves a good performance.

The molecule could be easily represented using a graph, so graph neural network (GNN) [20] is suitable for extracting feature of the molecule. GNN could obtain the local and global structural information by using neighbor node features to update feature. Through the transmission of multi-layer networks, the feature of the whole data can be extracted. It has been successfully applied in CPI and DTA prediction task. For example, Tsubaki et al. [21] develops a novel CPI prediction method by combining graph neural network (GNN) and convolution neural network (CNN) for compounds and proteins representation respectively. GraphDTA [22], MCN-CPI [23] and PADME [24] also construct graphs to describe molecules and apply GNN for the feature extraction in DTA prediction. The achievements of these methods demonstrate that the GNN could effectively characterize the small molecule.

Due to the simplicity of the molecule, most existing methods can effectively mine their structural information, but structural information contained in protein sequence is always ignored. Moreover, as we stated above, there are still many targets without structure. To obtain structural information from sequence, the commonly used method is protein structure prediction. After decades of development, the accuracy of protein structure prediction has gradually increased. Especially with the emergence of AlphaFold [25], a breakthrough has been made in this field. Protein structure prediction usually needs to predict the interaction between different residue pairs, which is called contact map. It is a two-dimensional matrix, in which each element in the matrix represents the distance or interaction probability between residues. Because proteins are formed by the interaction of residues, contact map can reflect the spatial structure of the whole protein. Thus, if the contact map can be obtained according to the sequence in a fast way, its structural information can be obtained more quickly, which is useful for the affinity prediction.

To improve the performance of DTA and CPI predictions, we proposed a sequence-based method using weighted graph neural networks, the contributions of this paper are listed as below:

- Protein structure is determined by its residue interaction, which is caused by molecular forces. Due to the different distance between residues, the interactions are different. Only using a binary value in contact map is not accurate to depict the interaction between residues. A weighted protein graph construction method is proposed in this paper, which could provide more detailed information of the residue interaction.
- In our previous work, we proposed DGraphDTA [26]model, which uses contact map to construct protein graph, and achieved good performance for DTA prediction. However, the MSA process used in DGraphDTA for structure prediction and feature generation is time-consuming. Protein structure prediction provides key information for CPI and DTA prediction, so how to select a fast and accurate method to improve the efficiency is another issue to be solved. In this work, the evolutionary scale modeling (ESM) [27] is used, which could keep the accuracy rate stable while significantly increasing the computing speed. Moreover, to solve the sequence length limitation, a subsequence merging method is proposed.
- Finally, a weighted graph neural networks model that could be suitable for the proposed weighted protein graph is established. Different experiments show that the proposed method has high accuracy for CPI and DTA prediction and is faster than our previous method.

## Results

We propose a novel method for protein sequence representation using weighted graph, and a model is constructed based on it, which is called as WGNN-DTA. In order to comprehensively test the accuracy of the proposed WGNN-DTA, various experiments are designed, including CPI and DTA prediction performance validation, contact map efficiency comparison.

### Datasets

Based on a variety of designed experiments, several datasets are involved. The datasets are described in detail as follows.

Davis and KIBA datasets: Davis [28] and KIBA [29] datasets are used to verify the DTA prediction performance of WGNN-DTA. It is a benchmark for DTA prediction used in DeepDTA [11] and is published. Davis dataset is obtained by screening some kinase proteins and their related inhibitors, and its binding affinity is the corresponding dissociation constant *K*_*d*_. The KIBA dataset is constructed from kinase family proteins and their inhibitors, and the binding KIBA score is calculated based on different affinity (*K*_*i*_,*K*_*d*_ and *IC*_50_). The detailed information of the two datasets shown in Table 1. For Davis dataset, the affinities are processed in the same way of DeepDTA, which is calculated using Eq. (1).

1 $$\begin{aligned} {pK}_{d}=-\log_{10}{\frac{K_{d}}{10^{9}}} \end{aligned}$$

**Table 1:** Davis and KIBA datasets

Dataset	Number of proteins	Number of drugs	Binding entries
Davis	442	68	30056
KIBA	229	2111	118254

Human and C.elegans datasets: the human and C.elegans of CPI datasets were created by Liu et al. [6] in 2015 and used by Masashi et al. [21] to verify the CPI prediction performance of their proposed method. The dataset includes highly credible negative samples of compound-protein pairs obtained by using a systematic screening framework. The positive samples of the dataset are obtained from two manually managed databases which are DrugBank [30] and matador [31]. The human dataset contains 3369 positive interactions between 1052 unique compounds and 852 unique proteins; The Caenorhabditis elegans (C.elegans) dataset contains 4000 positive interactions between 1434 unique compounds and 2504 unique proteins.

DUD-E dataset: DUD-E [32] is designed for benchmark molecular docking programs by providing challenging decoys. There are 102 targets in the dataset, and each target is provided with several active molecules and decoys, which constitutes the CPI dataset of positive samples and negative samples. The dataset contains the structure information of proteins, so it is used in our work to test whether the proposed WGNN-DTA can be extended to the structure-based prediction.

### Performance of compound-protein interaction (CPI) prediction with WGNN-DTA

Compound-protein interaction (CPI) prediction is an important task of virtual screening. In order to verify the performance of WGNN-DTA proposed in our work, two CPI prediction experiments are introduced.

**Fig. 1:**
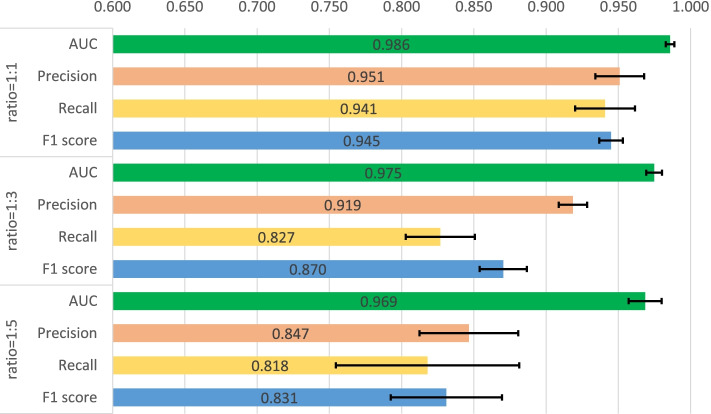
Five-fold cross validation of CPI prediction performance on human dataset with GCN implementation

**Fig. 2:**
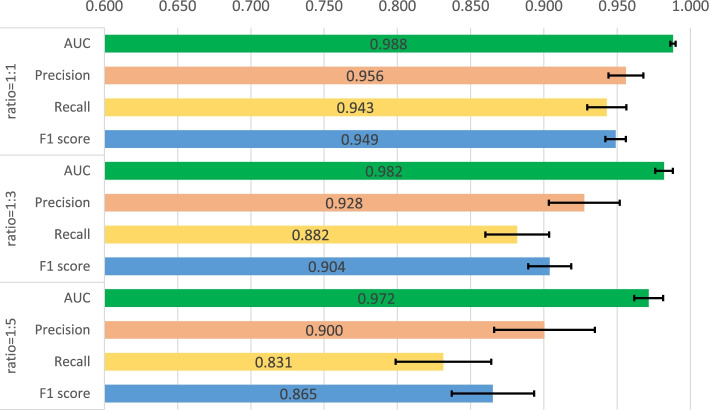
Five-fold cross validation of CPI prediction performance on human dataset with GAT implementation

**Fig. 3:**
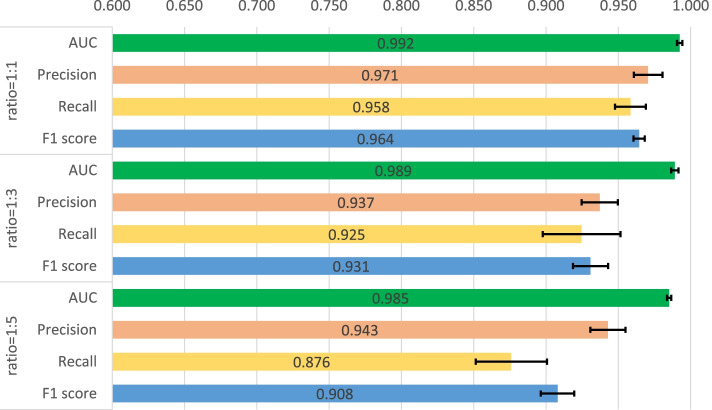
Five-fold cross validation of CPI prediction performance on C.elegans dataset with GCN implementation

**Fig. 4:**
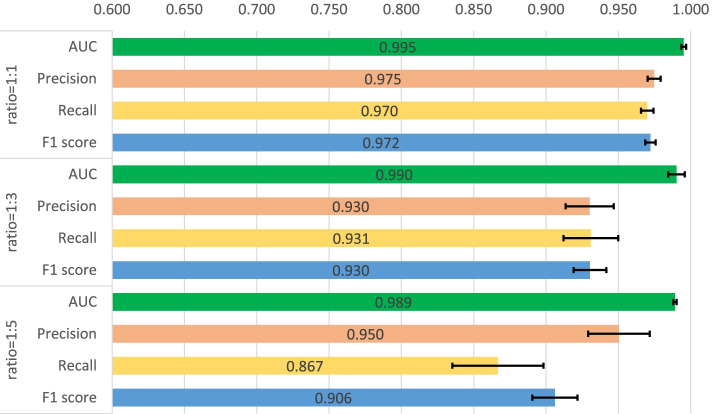
Five-fold cross validation of CPI prediction performance on C.elegans dataset with GAT implementation

First, a five-fold cross validation experiment is implemented on two datasets including human and C.elegans datasets [6], where every dataset is randomly divided into five parts with the same size and every part is used to verify the performance of the model trained by the other four parts in turn. In Masashi’s implementation [21], they verified the performances for their method with a ratio of 1:1, 1:3 and 1:5 for positive and negative samples. We also adopted the same setting, and the same measures are used for the performance comparison, which consists of area under curve (AUC), precision and recall. Precision and recall are commonly used measures to evaluate the binary classification, which are calculated through the Eqs. 2 and 3, where TP, FP and FN means the number of true positive predictions, false positive predictions and false negative predictions. In addition, F1-score is also involved to measure the proposed method which is calculated through Eq. 4. The used hyperparameters for our model are listed in Table 2 and the five-fold cross validation results for the two datasets are shown in Figs. 1, 2, 3, 4. The black line in the histogram indicates the standard deviation.

2 $$\begin{aligned} Precision=\frac{TP}{TP+FP} \end{aligned}$$

**Table 2:** Hyperparameters used in the experiment

Number	Name	Setting
1	The number of GNN layers	3
2	The type of GNN pooling layer	Global mean pooling
3	Activation function	Sigmoid
4	Optimizer	Adam
5	Learning rate	0.001
6	Loss function	Binary cross entropy loss
7	Epochs	1000
8	Batch size	512

3 $$\begin{aligned} Recall=\frac{TP}{TP+FN} \end{aligned}$$

4 $$\begin{aligned} F1-score=\frac{2\times(Precision+Recall)}{Precision \times Recall} \end{aligned}$$

The four figures illustrate that the performance of WGNN-DTA achieve a high performance whether using GCN or using GAT for the model construction, and the AUC measure on two datasets are all above 0.9 in the five-fold cross validation. When the ratio of positive and negative samples is 1:1, the performance is the best. With the proportion of negative samples increases, the performance decreases, and the standard deviation also increases gradually, which means that the stability of the model decreases. This is because unbalanced data will lead to poor fitting of the model. But even the ratio is 1:5, WGNN-DTA could still have a good capability, where the AUC measure reaches 0.969 and 0.985 with GCN implementation and reaches 0.972 and 0.989 with GAT implementation on the two datasets.

**Fig. 5:**
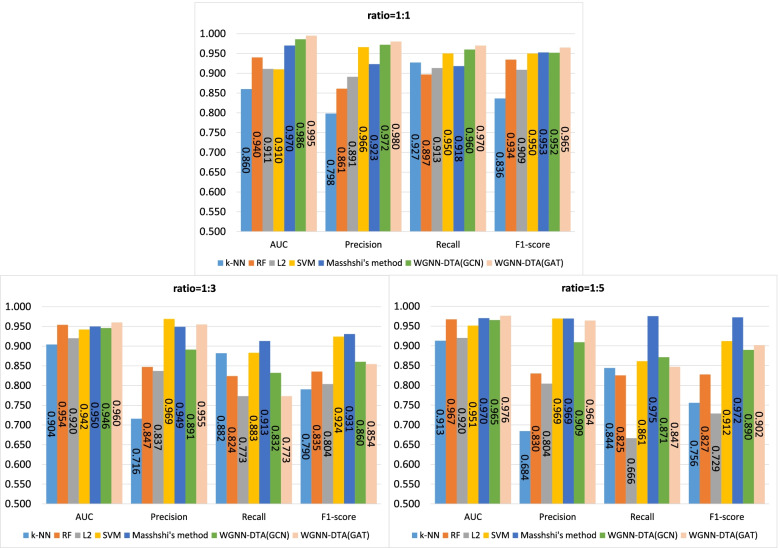
CPI prediction performance on human dataset

**Fig. 6:**
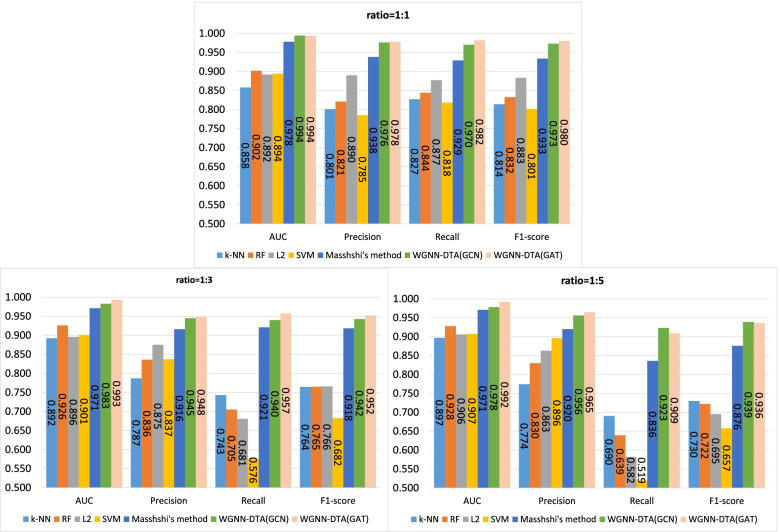
CPI prediction performance on C.elegans dataset

To further validate the performance of WGNN-DTA, Masashi’s method [21] is involved for comparison. The same CPI datasets as Masashi are used, and the same dataset division is implemented, in which the dataset is randomly divided into training set, validation set and test set with a proportion of 0.8, 0.1 and 0.1. The dataset settings with ratios of 1:1, 1:3 and 1:5 for positive and negative samples are also adopted, and the same measures are used for the performance comparison, which consists of AUC, recall and precision. In addition, F1-score measure is introduced. We compare WGNN-DTA with Masashi’s method and other traditional machine learning methods including k-NN, random forest (RF), L2 logistic (L2) and support vector machine (SVM), experimental results of which are referenced from the Masashi’s paper [21]. The comparison results are shown in Figs. 5 and 6.

It is illustrated from the figures that the performance of WGNN-DTA has a high accuracy on two CPI datasets. For the human dataset, WGNN-DTA achieve a better prediction on balanced data with AUC scores of 0.986 and 0.995, and the prediction performance declines on unbalanced data. But for C.elegans dataset, whether GCN or GAT is used, WGNN-DTA has a superior prediction performance on all ratios, which is better than other methods. The AUC scores are both more than 0.990 on balanced and unbalanced data with GAT implementation.

So it can be concluded from the experimental results that the performance of WGNN-DTA for CPI prediction is superior to other methods. This is because the structural information of proteins and small molecules are fully represented by constructing weighted protein graph and molecular graph, and through the mining of the GNN model, the factors affecting the binding can be accurately taken into account, which leads to a better prediction performance.

### Performance of drug-target affinity (DTA) prediction with WGNN-DTA

Compared with CPI prediction, the drug-target affinity (DTA) prediction is more complex. CPI prediction only needs to determine whether a molecule can bind the protein or not, while DTA prediction needs to predict the detailed binding affinity.

**Fig. 7:**
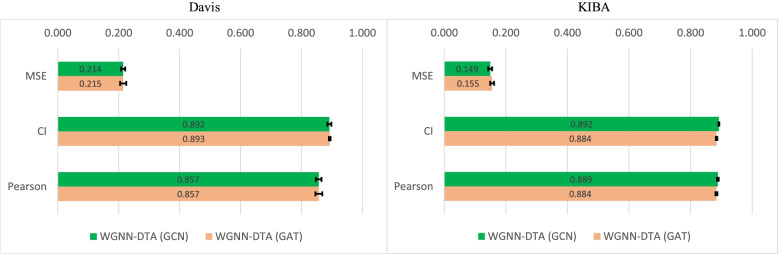
Five-fold cross validation of DTA prediction performance with WGNN-DTA on Davis and KIBA datasets

Similar to CPI experiment, a five-fold cross validation experiment is firstly implemented on Davis [28] and KIBA [29] datasets to test the stability of the model. The published datasets have already divide each dataset into five parts and one test part randomly, and the five parts in training set are used for the validation in the experiment. Mean square error (MSE), concordance index (CI) and the extra Pearson correlation coefficient (Pearson) are introduced for performance measurement, where a smaller MSE or a higher CI and Pearson of the results means a better performance of the model. The used hyperparameters are listed in Table 3 and the validation results are shown in Fig. 7. It is shown that WGNN-DTA with GCN implementation performs well on KIBA dataset, which could reach 0.149 with MSE measurement, the performance of GCN implementation and GAT implementation is almost the same on Davis dataset with a MSE of 0.214 and 0.215. In addition, the standard deviation is small, which means that WGNN-DTA has good stability on the two datasets.

**Table 3:** Some hyperparameters used in the experiment

Number	Name	Setting
1	The number of GNN layers	3
2	The type of GNN pooling layer	Global mean pooling
3	Activation function	Not used
4	optimizer	Adam
5	Learning rate	0.001
6	Loss function	Mean squared error loss
7	Epochs	2000
8	Batch size	512

**Fig. 8:**
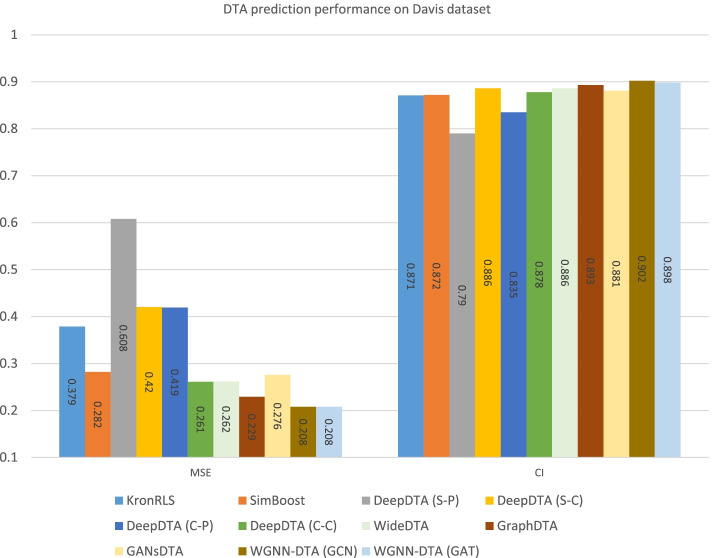
Comparison of DTA prediction performance on Davis dataset

**Fig. 9:**
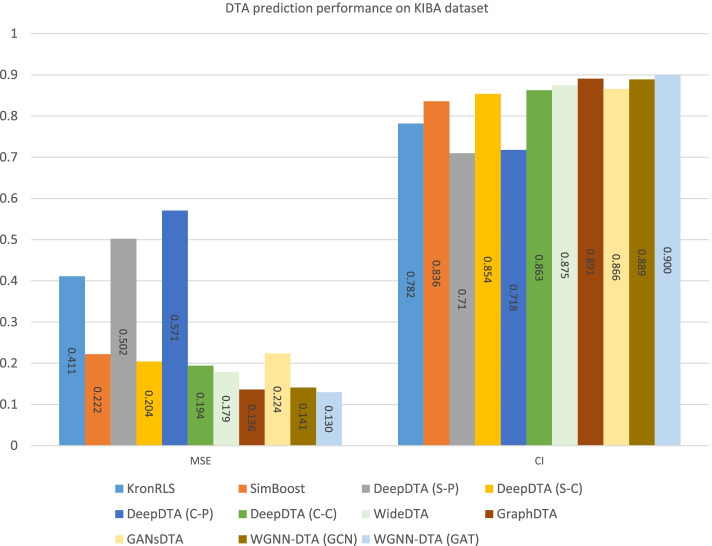
Comparison of DTA prediction performance on KIBA dataset

To comprehensively evaluate the performance of the proposed method on DTA prediction, we compare our work with DeepDTA [11], GraphDTA [22] and GANsDTA [14] using the same benchmark datasets including Davis [28] and KIBA [29] datasets. The same training set and test set are implemented, as well as the same performance measures are introduced for evaluation. All other method prediction results are referenced from their papers. The performance comparison is illustrated in Figs. 8 and 9.

Compared with other methods, the performance of DTA prediction is improved with WGNN-DTA. No matter using GCN or GAT, the proposed method has excellent performance with all measures used for evaluation. WGNN-DTA could reach 0.208 and 0.130 on Davis and KIBA datasets with MSE measure, which is smaller than other methods and suggests a better performance. In addition, the CI score of the predictions for WGNN-DTA is also higher than other methods, which indicates the comprehensively excellent performance of the model. Moreover, the Pearson correlation coefficient measure used in WideDTA is calculated to evaluate WGNN-DTA, which is illustrated in Table 4. It can be seen from the table that WGNN-DTA performs well with Pearson correlation coefficient measure evaluation.

**Table 4:** Performance of DTA prediction with Pearson correlation coefficient measure on two datasets

Method	Davis	KIBA
WideDTA	0.820	0.856
WGNN-DTA (GCN)	0.862	0.891
WGNN-DTA (GAT)	**0.863**	**0.900**

The values in boldface represent the best prediction performances with the corresponding measures

Molecular SMILES and protein sequence contain a wealth of structural information, especially for proteins, which include the function and binding site information. By constructing the graphs for proteins and drugs, these features can be encoded effectively, and the representations further mined by the GNN model. The hidden structural and binding sources can be fully obtained, which plays an important role in the final affinity prediction.

### Performance of edge weights

WGNN-DTA constructs a weighted protein graph based on the contact probability. It could show the interaction between different residues more accurately, and could comprehensively describe the protein structure. To demonstrate whether the added edge weights improve the prediction performance or not, the protein graphs are constructed with and without weights, and the performances of different graph construction methods are compared. In the experiment, CPI and DTA prediction are all involved, and Human [6] balanced (positive samples: negative samples= 1:1) dataset and Davis [28] dataset are used respectively. The prediction results are shown in Tables 5 and 6.

**Table 5:** Performance of CPI prediction with and without edge weights on human dataset

Method	AUC	Precision	Recall
WGNN-DTA (GCN)	0.986	0.972	0.960
WGNN-DTA (GAT)	**0.995**	**0.980**	**0.970**
Without Weight (GCN)	0.967	0.927	0.869
Without Weight (GAT)	0.970	0.930	0.877

The values in boldface represent the best prediction performances with the corresponding measures

**Table 6:** Performance of DTA prediction with and without edge weights on Davis dataset

Method	AUC	Precision	Recall
WGNN-DTA (GCN)	**0.208**	**0.902**	0.862
WGNN-DTA (GAT)	**0.208**	0.898	**0.863**
Without Weight (GCN)	0.215	0.893	0.856
Without Weight (GAT)	0.224	0.892	0.850

The values in boldface represent the best prediction performances with the corresponding measures

It is illustrated that the introduction of edge weights improved the prediction performance for both CPI and DTA prediction. In the folding structure of protein, even if the residues can generate an interaction, the interaction strength may be different, which is a reflection of molecular forces such as hydrogen bond. The closer the residues are, the more likely they can interact with each other. WGNN-DTA takes the probability prediction of interaction as the edge weight of the constructed protein graph, which can take the interaction strengths between different residues into account, thus it could represent the protein structure more comprehensively and thoroughly, and improves the binding prediction performance with small molecule.

### Performance of contact map prediction

The first step in the proposed method is to predict contact map according to the corresponding sequence, then the weighted protein graph can be constructed based on it. The performance of the contact map prediction will greatly influence the accuracy and efficiency of CPI and DTA prediction. Therefore, another experiment is set up to verify the contact map prediction performance and the speed of WGNN-DTA.

In our previous work, our proposed DGraphDTA uses PconsC4 [33] for the contact map prediction, which needs to implement the MSA for every protein at first. To compare the accuracy of WGNN-DTA and our previous MSA-based method in DTA prediction, the test set of Davis dataset is introduced for the comparison. The predictions of the two methods are run on a server with a single core mode of an Intel(R) Xeon(R) E5-2620 CPU and a GTX 1080Ti GPU. The performance of DGraphDTA and WGNN-DTA are shown in Table 7.

**Table 7:** Performance comparison of WGNN-DTA with DGraphDTA

Method	MSE	CI	Pearson
DGraphDTA	**0.202**	**0.904**	**0.867**
WGNN-DTA (GCN)	0.208	0.902	0.862

The values in boldface represent the best prediction performances with the corresponding measures

It can be seen from the table that the performance degradation of the proposed method is limited, which achieve an MSE of 0.208. Therefore, although WGNN-DTA removes the complex MSA processing, the constructed protein graph could still describe the structural information of protein effectively, which results in a high accuracy.

Moreover, two other methods are introduced for the speed comparison, which includes DeepDTA and GraphDTA. The prediction time of the two methods together with WGNN-DTA and DGraphDTA are calculated for the test set. The time includes the time of data processing and model prediction. The results are illustrated in Table 8.

**Table 8:** Speed comparison of WGNN-DTA with other methods

Method	Total time (min)
DGraphDTA	15900
WGNN-DTA (GCN)	50
DeepDTA	**0.5**
GrapDTA	2.5

The values in boldface represent the best prediction performances with the corresponding measures

It can be concluded that DeepDTA has a faster speed for the prediction. DeepDTA utilizes CNN directly to the SMILES and sequence, and there is no other additional process for the prediction. GraphDTA also encodes sequence using CNN, but it introduces molecular graph processing, which takes a little more time. WGNN-DTA construct graphs for both molecule and protein, especially when constructing weighted protein graph, the ESM model is implemented for contact map prediction, which takes up most of the time. Obtaining structural information requires more calculations, so WGNN-DTA takes more time on this to improve the accuracy. But even so, for the whole test set formed by more than 400 proteins, it only takes a total of 50 minutes. Compared with the previous DGraphDTA, it improves the efficiency greatly. MSA processing have to generate a list of similar sequences for the target, and the database searching is a large time consuming process. The proposed WGNN-DTA no longer needs MSA profiles as input, which saves a lot of time and still maintain high accuracy. Thus, WGNN-DTA could improves DTA prediction efficiency.

### Performance of contact map prediction

From the above experiments, it can be seen that the proposed WGNN-DTA has high performance for the sequence-based DTA and CPI prediction. In order to verify whether the proposed method can be extended to the structure-based prediction, an additional experiment is implemented on data with structural information, which could obtain their contact map directly. We construct the corresponding weighted protein graph based on its real contact map with a threshold of 8Å, and use the distance between different pair of residues as the edge weights. The WGNN-DTA is used for feature extraction to predict the binding interaction. In this experiment, DUD-E [32] is introduced for the validation. We select 200 active molecules and decoys for each protein, which yield total of around 35000 molecule-protein pairs, random 60% proteins (61 proteins) and their binding molecules and decoys are used as the training set and the remain 40% proteins (41 proteins) are used as the test set. The detailed experimental results are shown in Table 9.

**Table 9:** CPI prediction performance on DUD-E dataset

Method	Protein rep.	Compound rep.	AUC	Precision	Recall
WGNN-DTA (GCN)	GCN	GCN	0.963	0.949	0.789
WGNN-DTA (GAT)	GAT	GAT	0.962	0.896	0.867

It is illustrated that the when WGNN-DTA applying to structure-based CPI prediction, the performance still remains quite better, which can achieve 0.963 and 0.962 AUC scores with GCN and GAT. It is shown that the constructed weighted protein graph and molecular graph can indeed represent the binding information and structural information of proteins and small molecules.

## Discussion

In order to improve the performance of DTA prediction, the WGNN-DTA method is proposed in this paper, which could well characterize small molecule and protein sequence by constructing molecular graph and weighted protein graph based on contact map. By using GNN for further feature extraction, the obtained latent vectors can represent proteins and molecules in a more sufficient way. Moreover, the introduction of contact map prediction method, ESA model, which removes the complex multiple sequence alignment process, improves the efficiency of the method. So the proposed method could be applied to virtual screening of large datasets.

With the accumulation of actual biological data, many relevant databases used for simulation and validation have been published. With the help of deep learning to assist analysis, more accurate simulation can be realized. At present, there are many research fields and many excellent cases have emerged. In our work, a novel weight graph is constructed to represent the protein sequence, and the graph neural network is used to construct the model to realize the predictions of DTA and CPI. The corresponding graphs of small molecule SMILES and protein sequence constructed according to the proposed method can be input into the model in practical application, then the binding prediction result could be obtained, which provides a powerful mean for the virtual screening of target proteins and assists the discovery of lead compounds.

Based on various experiments, it is demonstrated that WGNN-DTA can not only be applied to DTA and CPI prediction, but also has good performance for the prediction extended to the structure-based prediction.

## Method

### WGNN-DTA architecture

**Fig. 10:**
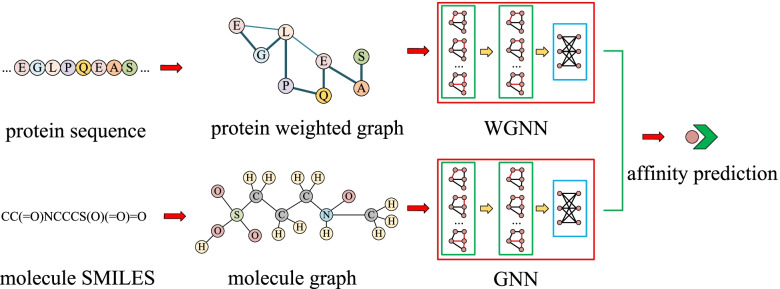
The process of WGNN-DTA for affinity prediction

The process of the proposed WGNN-DTA is shown in Fig. 10. The input are protein sequence and molecule SMILES. A weighted protein graph is constructed based on the contact map, which could comprehensively describe the protein structure. Instead of binary value used in DGraphDTA, the interaction probability is used as edge weight, which could provide more detailed structural information. In addition, molecular graph is introduced to describe molecule, the atoms are used as node and bonds are used as edge. Finally, a method called weighted GNN drug-target affinity predictor (WGNN-DTA) is proposed, which uses two types of GNNs including graph convolutional network (GCN) [34] and graph attention network (GAT) [35] to extract the latent vectors of protein and molecular graphs, and the affinity prediction can be achieved base on the latent vectors. Some node features that can be quickly generated are selected to improve the performance of the method.

### Weighted protein graph and latent vector extraction

The first important step is to construct weighted protein graph, which will directly influence the accuracy of the prediction. Protein sequence is a string composed of about 20 symbols, but it contains rich evolutionary and structure information. How to extract the information from the sequence is the key factor to promote the prediction accuracy.

Contact map is the result of protein structure prediction. There are many structure prediction methods, such as AlphaFold [25]. However, most existing structure prediction methods need to carry out sequence alignment by scanning the sequence database, which spends lots of time and greatly weaken the efficiency of affinity prediction. Therefore, the evolutionary scale modeling (ESM) model proposed by Rao et al. [27] is involved in our work for contact map prediction, which could obtain relatively accurate results without sequence alignment, and the prediction of a protein sequence can be completed in less than a minute. In addition, we verified that the contact prediction was relatively accurate for DTA prediction, and the detailed validation is described in the experiment part.

**Fig. 11:**
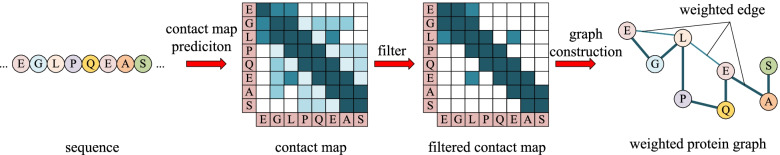
Construction of weighted protein graph

After obtaining the contact map, the weighted graph for protein is constructed. The obtained contact map is a probability matrix, which indicates the interaction probability of different residue pairs, and the range of probability is [0,1]. Normally, a threshold is used to convert the probability matrix to a binary matrix to indicate whether two residues are connected or not. The weighted protein graph is constructed according to contact map with residue as node and interaction as edge, the value of 0.5 is set as the threshold which means there will be an edge between residues with interaction probability exceeding 0.5. Different with the protein graph proposed in our previous DGraphDTA, we do not only use binary matrix as edge, but also use the probability value as the edge weight. The detailed construction process is shown in Fig. 11, the intensity of the contact map indicating the weight of the corresponding edge.

In the constructed weighted protein graph, the residue is used as the node, so it is need to describe the features for different residue nodes. The selected residue features are shown in Table 10. Due to the different R group of residues, the residues show different properties, and the properties further influence their interactions. It is noted that in order to speed up the processing, we remove the position-specific scoring matrix (PSSM) profile introduced in our previous work, because the PSSM needs to be calculated based on MSA processing.

**Table 10:** Residue node feature

Feature name	Feature description	Dimension
Residue type	One-hot encoding of the residue	21
Residue aliphatic	Whether the residue is aliphatic	1
Residue aromatic	Whether the residue is aromatic	1
Residue polar	Whether the residue is polar neutral	1
Residue Acidic polar	Whether the residue is acidic charged	1
Residue basic polar	Whether the residue is basic charged	1
Residue weight	The molecular weight of the residue	1
−*COOH* property	Dissociation constant for the −*COOH* group [36]	1
−*NH*_3_ property	Dissociation constant for the −*NH*_3_ group [36]	1
Other groups property	Dissociation constant for any other group in the molecule [36]	1
Residue isoelectric	The pH at the isoelectric point [36]	1
Hydrophobicity 1	Hydrophobicity of residue (pH = 2) [37]	1
Hydrophobicity 2	Hydrophobicity of residue (pH = 7) [37]	1
All	All features of the residue	33

**Fig. 12:**
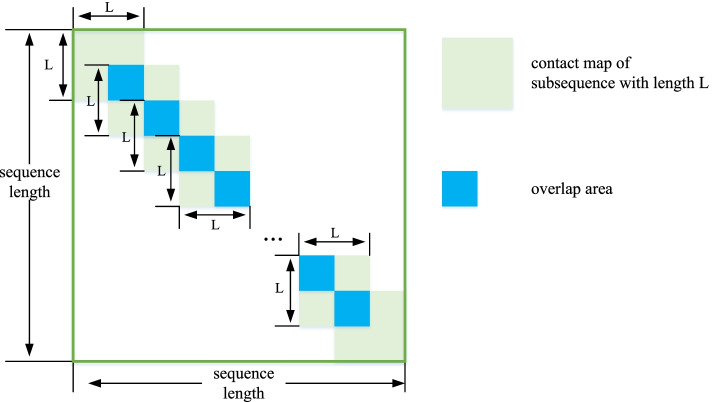
Intercepting and splicing of the contact map prediction for large proteins (with a sequence length over 1000)

The length of input sequence of ESM model is required to be within 1024, so it is need to find a way to deal with longer sequences. It is well-known that the close residues in the sequence usually generate an interaction, the value on the diagonal of contact map is what we focus on. Thus, based on the above observations, for proteins with long sequence (more than 1000), we use intercepting and splicing to reconstruct the contact map, and the process is shown in Fig. 12. The whole sequence is cut into multiple subsequences, the length and step of which is set as L (set as 1000 in our work) and L/2. The contact map of each subsequence is calculated in turn, and the final contact map is reconstructed according to them. For the overlapping part of the subsequence, the contact map is obtained by averaging them (blue area in Fig. 12). Through experiment verification, we find that the contact map obtained by this method can still maintain high accuracy.

In addition, since adjacent residues are connected by peptide bonds in principle, the value of any adjacent residues is set to 1, and a self-loop is also added, which means each residue connect with itself.

The weighted protein graph can initially represent the proteins, so a neural network needs to be established to deeply mine the latent feature. Graph neural network (GNN) is applied to extract the features of the constructed graph in WGNN-DTA. GNN is a deep learning model gradually used in recent years. The traditional convolution neural network can only extract the features of Euclidean structure data with fixed sizes, which limits its application. But GNN could handle non-Euclidean structure data such as graph, which ensure it could be widely used. The commonly used GNN models include graph convolution neural network (GCN) and graph attention network (GAT), which is also introduced to establish the proposed WGNN-DTA.

For GCN, each layer will execute the following equation:

5 $$\begin{aligned} H^{l+1}=f\left(H^{l},A\right)=\sigma\left(\hat{D}^{-\frac{1}{2}}\hat{A}\hat{D}^{-\frac{1}{2}}H^{l}W^{l+1}\right) \end{aligned}$$

where *A* is the adjacency matrix of the graph with shape of (*n*,*n*), and *n* is the number of nodes of the graph. $\hat {A}=A+I$, and *I* is the identity matrix. $\hat {D}$ is the degree matrix calculated according to *A*, which has the same shape as *A*. *W*^*l*+1^ is the weight matrix of layer *l*+1, and it can be learned during training. *H*^*l*^ is the output of the last layer with a shape of (*n*,*F*^*l*^). *F*^*l*^ is the number of output channels of layer *l*, and *H*^0^=*X*, *H*^0^=*X* is the input feature matrix of the graph node.

The node features of each layer for GAT are calculated as:

6 $$\begin{aligned} h_{i}=\sigma\left(\sum\limits_{j\in N(i)}\alpha_{ij}{WX}_{j}\right) \end{aligned}$$

7 $$\begin{aligned} \alpha_{ij}=\frac{e^{a\left(h_{i},h_{j}\right)}}{\sum\limits_{k\in N(i)}a(h_{k}h_{i})} \end{aligned}$$

where, *N*(*i*) is the set of neighbor nodes of node *i*, *W* is the weight matrix, and *X*_*j*_ is the input feature matrix of node *j*, *α*_*ij*_ is the attention coefficient after regularization through Eq. (7). *a*(*x*) is a mapping function $R^{F^{l}}\times R^{F^{l}}\rightarrow R$, which can calculate the non-regularization coefficients of a pair of node *i* and *j*.

**Fig. 13:**
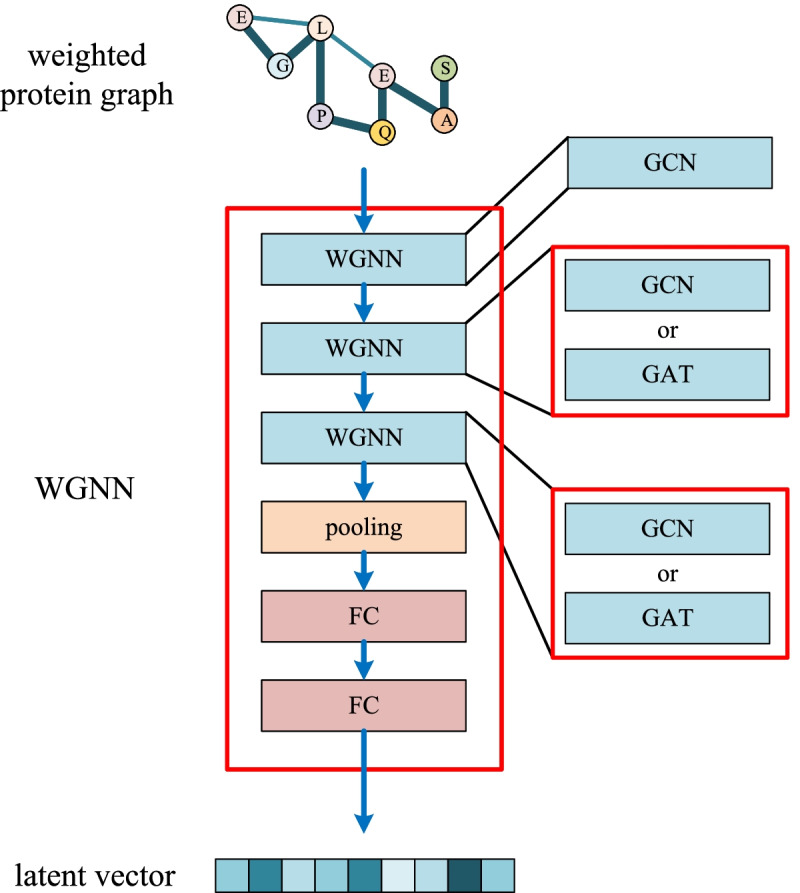
The detail neural network using for weighted protein graph feature extraction in WGNN-DTA

The detailed WGNN-DTA structure is shown in Fig. 13. Because the contact probability is used as the edge weight in the construction of weighted protein graph, weighed GNN is used to extract protein features. In implementation, when using GAT to establish WGNN-DTA, it is difficult for GAT to involve the weights, so we first use a layer of GCN to obtain the weight information of the graph, and then use GAT layers for the further construction of the model.

There is rich structure information in protein sequence, which determines its function and contains the mechanism of drug-target binding. The protein structure is then determined by the residue interactions, and the proposed weighted protein graph can present the residue interaction in the way of edge weight, which effectively shows the hidden structure information of protein. At the same time, the properties of residue will be represented in the form of node features, which can achieve a more accurate and comprehensive sequence representation.

### Molecular graph and latent vector extraction

**Fig. 14:**
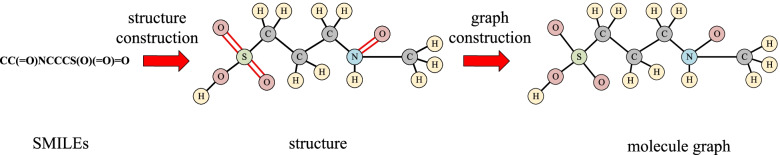
Construction of molecular graph

In sequence-based affinity prediction, molecule is usually represented by SMILES, which could convert the original atoms and covalent bonds into an ASCII character sequence. It is easy to restore SMILES back to the form of molecular structure with common molecular processing softwares such as RDKit [38]. Then the molecular graph is constructed with atom as node and chemical bond as edge. More specifically, when constructing the molecular graph, all atoms in the molecule are set as graph nodes, and if there is a bond between two atoms, then an edge will be added between the corresponding atom nodes. The construction process is shown in Fig. 14. Moreover, in the same way as the construction of protein graph, self-loop is also introduced when constructing molecular graph, in which each atom node is connected to itself.

Similar to protein graph construction, in addition to nodes and edges, the features of atom nodes should be described to distinguish them. So it is necessary to determine the node feature for different atoms. Atoms are the smallest particles in chemical reaction. Due to their different sizes and charges, different atoms show different chemical properties. The selected features of atom node are shown in Table 11. By characterizing the features of different atom nodes, the chemical properties and binding properties of small molecule can be expressed more comprehensively. Therefore, the factors influencing the binding of molecule is involved, which can promote the prediction performance.

**Table 11:** Atom node feature

Feature name	Feature description	Dimension
Atom type	One-hot encoding of the atom	44
Atom neighbors	One-hot encoding of the degree of the atom in the molecule, which is the number of directly-bonded neighbors	11
Number of hydrogens	One-hot encoding of the total number of H bound to the atom	11
Number of implicit hydrogens	One-hot encoding of the number of implicit H bound to the atom	11
All	All features of the atom	78

GCN or GAT is also utilized to extract features after the molecular graph is constructed, and processed by global pooling, molecules with different sizes are extracted into latent vectors with the same size. The connections and atom features will eventually influence the molecule properties and its binding with the target protein.

**Fig. 15:**
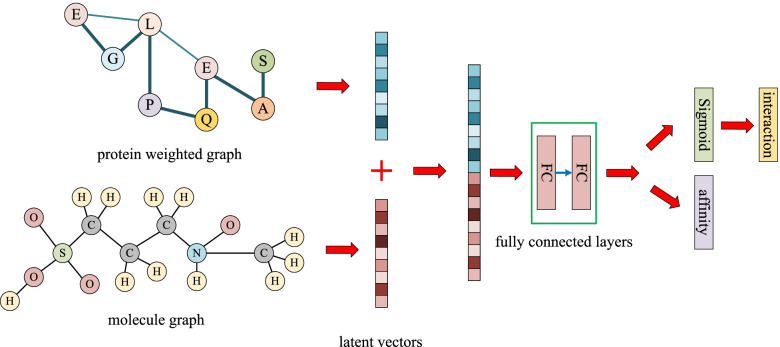
Affinity and interaction prediction in WGNN-DTA

After feature extraction of GNN, the latent vectors of the weighted protein graph and molecular graph are obtained. Then the two latent vectors are concatenated and further extracted by the neural network to realize the final binding prediction, which is illustrated in Fig. 15. Whether it is used for CPI or DTA prediction, WGNN-DTA can be competent. The only difference is whether to add a sigmoid activation function to the output.

At the same time, because the contact map prediction does not need sequence alignment and the generation of node feature can be quickly calculated by sequences, the prediction is fast and easy to be implemented. Thus, the proposed WGNN-DTA is suitable for the virtual screening of large databases, which improves the efficiency of sequence-based DTA prediction.

## Data Availability

The code and data are provided at https://github.com/595693085/WGNN-DTA.

## Abbreviations

DTA: Drug-target affinity
CPI: Compound-protein interaction
CNN: Convolutional neural network
GNN: Graph neural network
WGNN-DTA: Weighted graph neural work drug-target affinity predictor
GCN: Graph convolutional network
GAT: Graph attention network
SMILES: Simplified molecular input line entry specification
AUC: Area under curve
MSE: Mean square error
CI: Concordance index
MSA: Multiple sequence alignment
